# Berberine Improves Cancer-Derived Myocardial Impairment in Experimental Cachexia Models by Targeting High-Mobility Group Box-1

**DOI:** 10.3390/ijms25094735

**Published:** 2024-04-26

**Authors:** Kei Goto, Rina Fujiwara-Tani, Shota Nukaga, Yoshihiro Miyagawa, Isao Kawahara, Ryoichi Nishida, Ayaka Ikemoto, Rika Sasaki, Ruiko Ogata, Shingo Kishi, Yi Luo, Kiyomu Fujii, Hitoshi Ohmori, Hiroki Kuniyasu

**Affiliations:** 1 Department of Molecular Pathology, Nara Medical University, 840 Shijo-cho, Kashihara 634-8521, Nara, Japan; ilgfgtk@gmail.com (K.G.); shota.nukaga@gmail.com (S.N.); y.miya1103@gmail.com (Y.M.); isao_kawahara@a011.broada.jp (I.K.); g.m__r1@outlook.jp (R.N.); a.ikemoto.0916@gmail.com (A.I.); rika0st1113v726296v@icloud.com (R.S.); pkuma.og824@gmail.com (R.O.); lynantong@hotmail.com (Y.L.); toto1999-dreamtheater2006-sms@nifty.com (K.F.); brahmus73@hotmail.com (H.O.); 2 Pathology Laboratory, Research Institute, Tokushukai Nozaki Hospital, 2-10-50 Tanigawa, Daito 574-0074, Osaka, Japan; nmu6429@yahoo.co.jp

**Keywords:** cancer cachexia, cardiac disorder, HMGB1, RAGE, berberine

## Abstract

Cardiac disorders in cancer patients pose significant challenges to disease prognosis. While it has been established that these disorders are linked to cancer cells, the precise underlying mechanisms remain elusive. In this study, we investigated the impact of cancerous ascites from the rat colonic carcinoma cell line RCN9 on H9c2 cardiomyoblast cells. We found that the ascites reduced mitochondrial volume, increased oxidative stress, and decreased membrane potential in the cardiomyoblast cells, leading to apoptosis and autophagy. Although the ascites fluid contained a substantial amount of high-mobility group box-1 (HMGB1), we observed that neutralizing HMGB1 with a specific antibody mitigated the damage inflicted on myocardial cells. Our mechanistic investigations revealed that HMGB1 activated both nuclear factor κB and phosphoinositide 3-kinases-AKT signals through HMGB1 receptors, namely the receptor for advanced glycation end products and toll-like receptor-4, thereby promoting apoptosis and autophagy. In contrast, treatment with berberine (BBR) induced the expression of miR-181c-5p and miR-340-5p while suppressing HMGB1 expression in RCN9 cells. Furthermore, BBR reduced HMGB1 receptor expression in cardiomyocytes, consequently mitigating HMGB1-induced damage. We validated the myocardial protective effects of BBR in a cachectic rat model. These findings underscore the strong association between HMGB1 and cancer cachexia, highlighting BBR as a promising therapeutic agent for myocardial protection through HMGB1 suppression and modulation of the signaling system.

## 1. Introduction

Cachexia is estimated to occur in 40–80% of patients with advanced cancer [1,2], accounting for 20–30% of all cancer-related deaths [3]. Decreased muscle mass reduces treatment tolerability and worsens disease prognosis [4,5]. Skeletal muscle catabolism and atrophy, or sarcopenia, is the most specific phenotype of cachexia [1,2,3,4,5]. Recently, damage to the same muscle tissue, the heart muscle, has begun to be recognized as an important problem. Weight loss is also associated with myocardial atrophy in patients with cachexia [6,7]. The risk of heart failure in cancer survivors is more than 1.5 times that in patients without cancer [8]. Heart failure is the second leading cause of death in patients with cancer after death from the cancer itself [9,10].

Most myocardial disorders in cancer patients are secondary to treatments such as anticancer drugs and are called cancer therapy-related cardiac dysfunction (CTRCD) [11]. CTRCD occurs in 10% of cancer patients receiving chemotherapy [12]. CTRCD is well known to occur with anthracycline anticancer drugs such as doxorubicin, but it also occurs with other chemotherapeutic agents and molecularly targeted drugs [13]. In contrast, it has been shown that advanced cancer causes functional and morphological disorders and heart failure regardless of the presence or absence of anticancer drug treatment [14,15]. We have also reported myocardial damage caused by cancer cells in mouse cachexia models [16,17]. Furthermore, analysis of autopsy cases has revealed that cardiac dysfunction is observed even in patients with cancer cachexia who are not receiving anticancer drug treatment [18]. However, the cause is not completely clarified. In this study, we will elucidate the cause of myocardial damage caused by cancer cells.

High-mobility group box-1 (HMGB1) is secreted by cancer cells and plays a pivotal role in promoting their proliferation, invasion, and metastasis by interacting with the receptor for advanced glycation end products (RAGE) expressed in cancer cells [19]. Additionally, HMGB1 binds to both RAGE and toll-like receptor-4 (TLR4) in skeletal muscle cells, thereby promoting skeletal muscle autophagy and ultimately leading to muscle atrophy [20]. Importantly, skeletal muscle atrophy has been observed to correlate with blood HMGB1 concentrations in human cancer cachexia cases [21]. Notably, studies have demonstrated that suppressing HMGB1 can mitigate myocardial damage [22,23].

Colorectal cancer (CRC) stands as the foremost cause of cancer-related mortality globally, with its incidence witnessing a recent surge [24]. In Japan, CRC claims the top spot in cancer incidence and ranks second in cancer-related fatalities [25]. Despite an average survival rate of 72.6% for CRC cases, the 5-year survival rate drastically declines to 18.8% for stage 4 cases marked by distant metastases [25]. Over half of patients with CRC succumb to cancer cachexia [26]. Notably, CRC elevates HMGB1 secretion levels, correlating with cancer progression severity [20]. Through its receptor RAGE, HMGB1 fosters CRC proliferation, invasion, and metastasis. Additionally, it triggers monocyte apoptosis, suppresses host antitumor immunity, and promotes liver metastasis [19]. While HMGB1 secreted by cancer cells is implicated in inducing cachexia in CRC patients, its link to myocardial damage remains elusive. This study aims to unravel the association between myocardial damage and HMGB1 expression in CRC.

Berberine (BBR), a plant alkaloid [27], plays a pivotal role in modulating intestinal immunity and reducing inflammatory cytokine levels [28], thus fostering normalization of the intestinal flora [29]. Our previous studies have reported that BBR induces cell death in gastrointestinal cancer and suppresses cancer stem cells [30]. Furthermore, BBR shows its potential by suppressing HMGB1, thereby eliciting anti-inflammatory responses across various diseases [31,32,33]. In this study, we delve into BBR’s impact on cancer-induced myocardial damage.

## 2. Results

### 2.1. Impact of Rat Cancerous Ascites on Myocardial Cells

**Figure 1:**
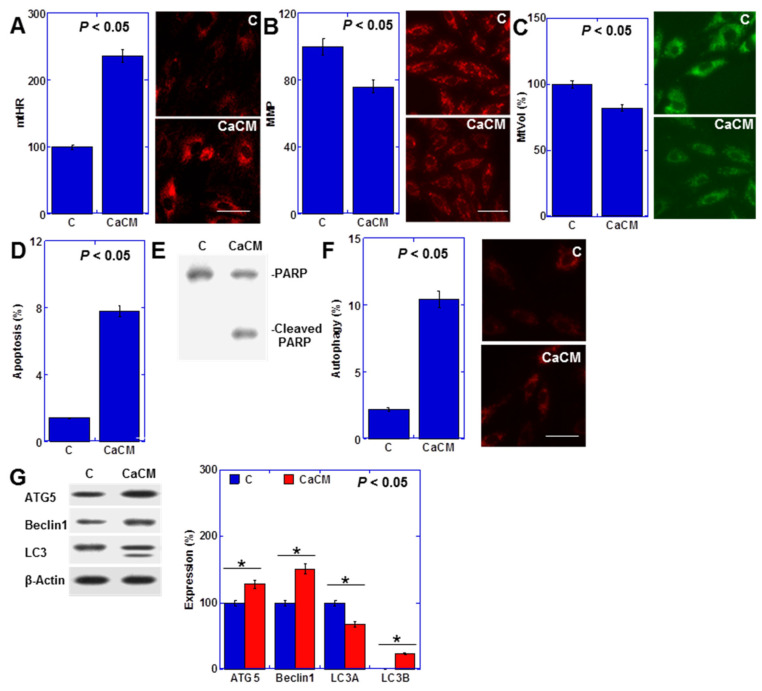
Effects of cancerous ascites fluid on rat H9c2 cardiomyoblasts. H9c2 cells were treated with CM containing ascites obtained from RCN9 CRC cells for 48 h. (**A**) MtHRs. (**B**) MMP. (**C**) MtVol. (**D**) Number of apoptotic cells. (**E**) PARP cleavage. (**F**) autophagy. (**G**) Levels of the autophagy-associated proteins. Right panels: fluorescent images (**A**–**C**,**F**); semi-quantified graph (**G**). Scale bar, 50 μm. Statistical significance was calculated using an ordinary ANOVA. Asterisk, *p* value. CaCM, cancerous ascites mixed culture medium; C, control medium; PARP, poly ADP-ribose polymerase; ATG5, autophagy-related gene 5; LC3, microtubule-associated protein 1A/1B-light chain 3; mtHR, mitochondrial hydroxylase; MMP, mitochondrial membrane potential; mtVol, mitochondrial volume.

Previously, we reported that the culture medium supplemented with rat cancerous ascites induces metabolic disorders similar to cachexia in cultured cardiomyocytes [17]. Employing a similar model, we observed an elevation in mitochondrial hydroxyl radical (mtHR) levels, alongside reductions in mitochondrial membrane potential (MMP) and mitochondrial volume (mtVol) (Figure 1A–C). Additionally, this condition triggered cardiomyocyte apoptosis (Hoechst 33342 staining and PARP cleavage) and autophagy (fluorescent staining and autophagy-associated protein levels) (Figure 1D–G).

### 2.2. Inflammatory Cytokines in Cancerous Ascites

**Figure 2:**
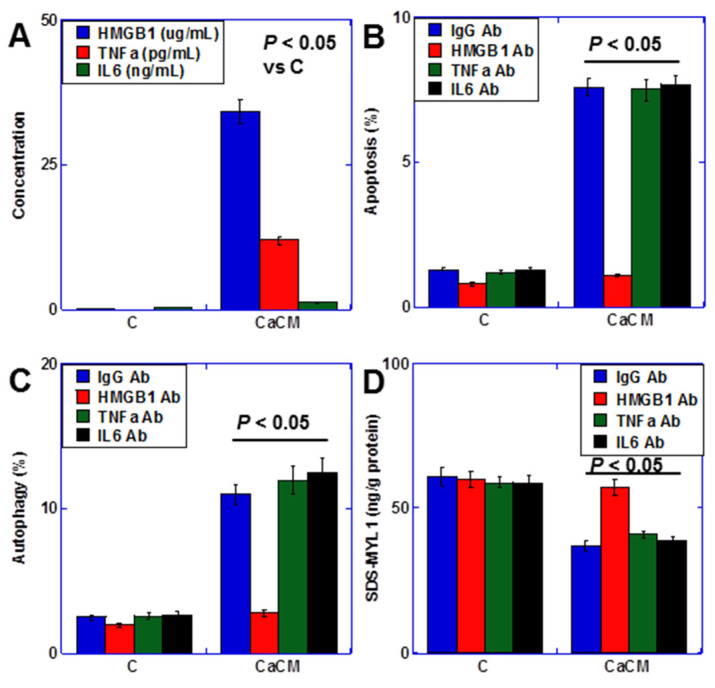
Inflammatory cytokines in rat cancerous ascites (**A**) Concentration of inflammatory cytokines. (**B**–**D**) Effect of anti-cytokine antibodies on apoptosis (**B**), autophagy (**C**), and myocardial maturation (SDS-MYL1) (**D**). Statistical significance was calculated using an ordinary ANOVA. HMGB1, high-mobility group box-1; TNF, tumor necrosis factor; IL, interleukin; CaCM, cancerous ascites mixed culture medium; C, control medium; Ab, antibody; SDS-MYL1, sodium dodecyl sulfate-soluble myosin light chain-1.

Inflammatory cytokines play a crucial role in cachexia [34,35]. Thus, we assessed the concentrations of HMGB1, tumor necrosis factor-α (TNFα), and interleukin-6 (IL6)—the primary inflammatory cytokines associated with cachexia—in the cancerous ascites utilized for cardiomyocyte treatment (CaCM) (Figure 2A). While all cytokines were present in the ascites fluid, HMGB1 was notably abundant. Consequently, we investigated the impact of these cytokines using neutralizing antibodies (Figure 2B,C). Remarkably, neutralizing HMGB1 exhibited a more pronounced reduction in both apoptosis and autophagy compared to TNFα and IL6. Moreover, the concentration of SDS-soluble myosin light chain 1 (SDS-MTL1), a marker of myocardial maturity [17], was also diminished by HMGB1 (Figure 2D). Hence, HMGB1 emerges as a key player in cancer-induced myocardial damage.

### 2.3. Effect of HMGB1 on Cardiomyocytes

**Figure 3:**
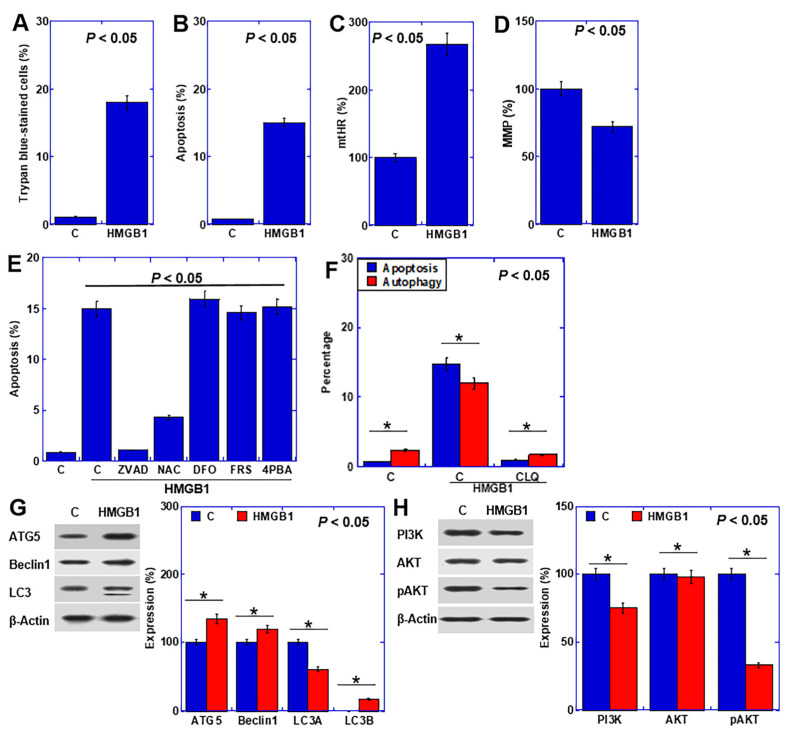
Effects of HMGB1 on rat H9c2 cardiomyoblasts. (**A**–**D**) Effect of HMGB1 on cell death (trypan blue staining) (**A**), apoptosis (**B**), mtHR (**C**), and MMP (**D**). (**E**) Inhibition of cell death (**F**) The effect of chloroquine on HMGB1-induced apoptosis and autophagy. (**G**,**H**) Effects of HMGB1 on the levels of autophagy-associated proteins (**G**) and autophagy signal-associated proteins (**H**). Right panels, semi-quantified graphs. Statistical significance was calculated using an ordinary ANOVA. Asterisk, *p* value. C, control; HMGB1, high-mobility group box-1 (40 μg/mL); mtHR, mitochondrial hydroxyl radical; MMP, mitochondrial membrane potential; ZVAD, Z-VAD-FMK; NAC, N-acetyl-L-cysteine; DFO, deferoxamine; FRS, ferrostatin-1; 4PBA, 4-phenylbutyric acid; ATG5, autophagy-Related Gene 5; LC3, microtubule-associated protein 1A/1B-light chain 3; PI3K, phosphoinositide 3-kinase; pAKT, phosphorylated AKT; CLQ, chloroquine.

To explore the impact of HMGB1 on cardiomyocytes, we subjected human recombinant HMGB1 to cardiomyocyte examination (Figure 3). HMGB1 treatment elicited increased cell death and apoptosis (Figure 3A,B), accompanied by an elevation in mtHR levels and a reduction in MMP (Figure 3C,D). In the cell death inhibitor assay, the apoptosis inhibitor ZVAD and the antioxidant N-acetylcysteine (NAC) effectively mitigated cardiomyocyte death (Figure 3E). Moreover, inhibition of HMGB1-induced autophagy by chloroquine resulted in a concomitant inhibition of apoptosis (Figure 3F). HMGB1 also upregulated autophagy-related proteins in cardiomyocytes (Figure 3G), while downregulating the autophagy inhibitory signals of phosphoinositide 3-kinases (PI3K) and phosphorylated AKT (Figure 3H).

### 2.4. HMGB1 Signal in Cardiomyocytes

**Figure 4:**
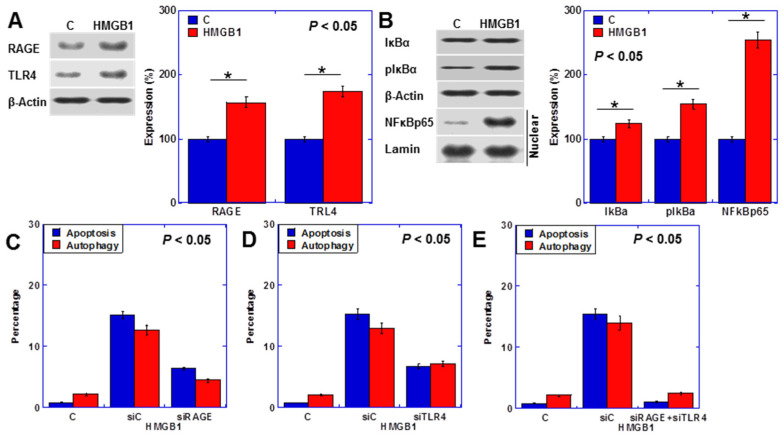
HMGB1 intracellular signal in H9c2 cells (**A**,**B**) Levels of HMGB1 receptor proteins (**A**) and NFkB signal-associated proteins (**B**). Right panels, semi-quantified graphs. (**C**–**E**) Effects of RAGE and/or TLR4 knockdown on apoptosis and autophagy. Statistical significance was calculated using an ordinary ANOVA. Asterisk, *p* value. C, control; HMGB1, high-mobility group box-1 (40 μg/mL); RAGE, the receptor for advanced glycation end products; TLR4, toll-like receptor 4; IκBα, nuclear factor of kappa light polypeptide gene enhancer in B-cells inhibitor alpha; pIκBα, phosphorylated IκBα; NFκBp65, nuclear factor of kappa light polypeptide gene enhancer in B-cells; C, control; siC, control short interferent RNA; siRAGE, short interferent RNA to RAGE; siTLR4, short interferent RNA to TLR4.

HMGB1 binds RAGE and TLR4 [20]. Although their expression was observed even without treatment, it increased upon HMGB1 treatment (Figure 4A). The nuclear factor-κB (NFκB) pathway, a common intracellular signal for RAGE and TLR4, was upregulated by HMGB1 (Figure 4B). Partial rescue of apoptosis (Hoechst 33342 staining) and autophagy (fluorescent staining) was achieved upon RAGE or TLR4 knockdown (Figure 4C,D). Conversely, the simultaneous knockdown of both RAGE and TLR4 restored apoptosis and autophagy to nearly normal levels (Figure 4E).

### 2.5. Effect of BBR on CRC Cancer Cells

**Figure 5:**
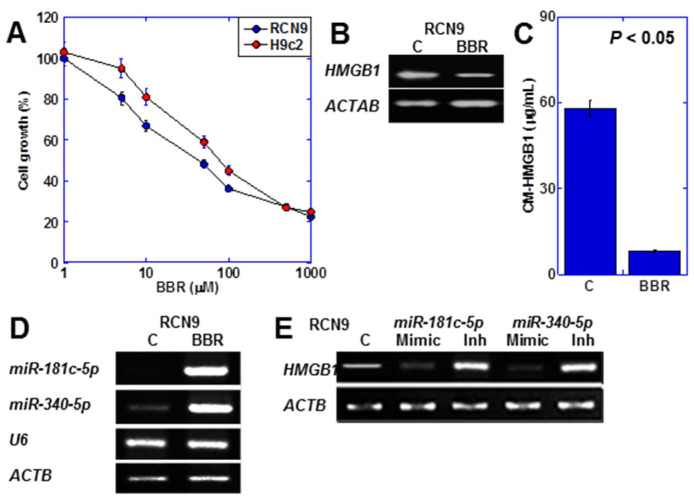
Effects of BBR on RCN9 rat CRC cells. (**A**) Effects of BBR on the growth of H9c2 and RCN9 cells. (**B**,**C**) Effects of BBR on HMGB1 mRNA expression (**B**) and HMGB1 protein in the culture medium (**C**). (**D**) Effects of BBR on miRNA expression. (**E**) Effects of miRNA mimics or inhibitors on HMGB1 expression. Statistical significance was calculated using an ordinary ANOVA. HMGB1, HMGB1, high mobility group box-1; ACTB, β-actin; C, control; BBR, berberine; U6, U6 small nuclear 1; mimic, microRNA mimic; Inh, microRNA inhibitor; CM-HMGB1, HMGB1 in the culture medium.

Subsequently, we investigated the impact of BBR on CRC cells secreting HMGB1 (Figure 5). We compared the inhibitory effect of BBR on the proliferation of H9c2 rat cardiomyocytes and RCN9 rat colon cancer cells (Figure 5A). The IC50 was 108 μM for H9c2 and 45 μM for RCN9. BBR suppressed HMGB1 expression in RCN9 cells (Figure 5B), resulting in approximately a sixfold reduction in HMGB1 concentration in the culture medium (Figure 5C). Moreover, BBR increased the expression of miR-181c-5p [36] and miR-340-5p [37], which are miRNAs known to suppress HMGB1 expression (Figure 5D). Subsequently, we examined HMGB1 expression using miRNA mimics and inhibitors (Figure 5E). The mimics nearly abolished HMGB1 expression, while the inhibitors led to an increase in HMGB1 expression for both miRNAs.

### 2.6. Effect of BBR on Cardiomyocytes

**Figure 6:**
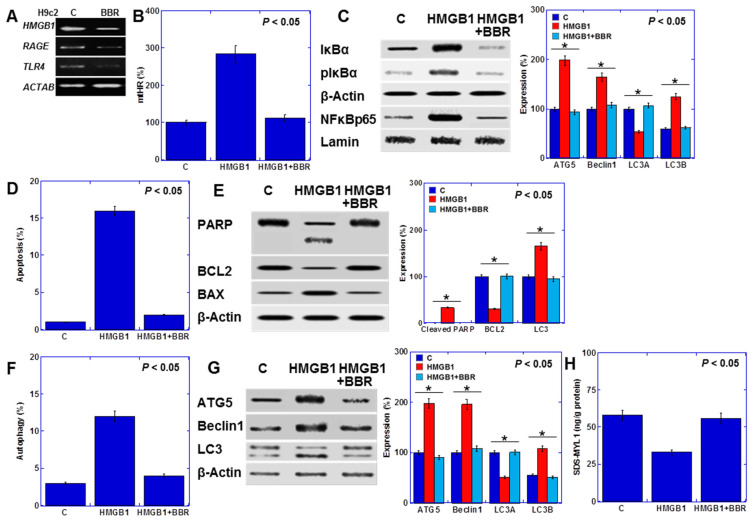
Efforts of BBR on H9c2 cells. (**A**) Effect of BBR on HMGB1 expression (**B**,**C**) Effect of BBR on HMGB1-induced mtHR (**B**), and NFkB signal proteins (**C**). Right panels, semi-quantified graph. (**D**,**E**) Effect of BBR on apoptosis (**D**) and the levels of apoptosis-associated proteins (**E**). Right panels, semi-quantified graph. (**F**,**G**) Effect of BBR A on autophagy (**F**) and the levels of autophagy-associated proteins (**G**). Right panels, semi-quantified graph. (**H**) Effects of BBR on myocardial maturation. Statistical significance was calculated using an ordinary ANOVA. Asterisk, *p* value. C, control; BBR, berberine; HMGB1, high mobility group box-1; RAGE, receptor for advanced glycation end products; TLR4, toll-like receptor 4; mtHR, mitochondrial hydroxyl radical; IκBα, nuclear factor of kappa light polypeptide gene enhancer in B-cells inhibitor alpha; pIκBα, phosphorylated IκBα; NFκBp65, nuclear factor of kappa light polypeptide gene enhancer in B-cells; PARP, poly ADP-ribose polymerase; BCL2, B-cell/CLL lymphoma 2; BAX, Bcl-2-associated X protein; ATG5, autophagy-Related Gene 5; LC3, microtubule-associated protein 1A/1B-light chain 3; SDS-MYL1, sodium dodecyl sulfate-soluble myosin light chain-1.

Next, we examined the effects of BBR on H9c2 cells (Figure 6). BBR reduced the expression of the HMGB1 receptors RAGE and TLR4 (Figure 6A). It also attenuated the induction of mtHR by HMGB1 (Figure 6B) and suppressed NFκB activation (Figure 6C). BBR mitigated the apoptosis induction by HMGB1 (Hoechst 33342 staining) (Figure 6D) while upregulating B-cell/CLL lymphoma 2 (BCL2) expression and downregulating Bcl-2-associated X protein (BAX) expression (Figure 6E). Furthermore, BBR abolished the promotion of autophagy by HMGB1 (fluorescent staining) (Figure 6F), leading to a decrease in the expression of autophagy-related genes and AKT phosphorylation (Figure 6G). Additionally, BBR reversed the HMGB1-induced reduction in myocardial maturity (SDS-MYL1) (Figure 6H).

### 2.7. Effect of BBR on Myocardial Damage Using a Rat Cancer Cachexia Model

**Figure 7:**
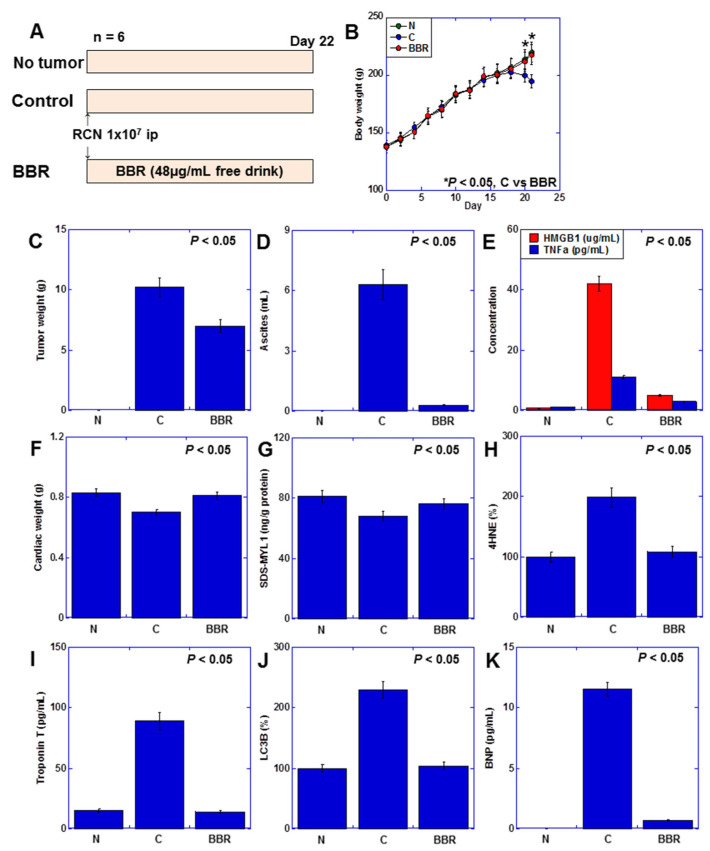
Effects of BBR on myocardial damage in a rat cancer cachexia model (**A**) Experimental protocol. F344 male rats (5 weeks old, 5 rats each group, SLC Japan, Shizuoka, Japan) were inoculated with RCN9 cancer cells (1 × 10^7^) intraperitoneally with or without administration of BBR (48 μg/mL, free drink). (**B**) Body weight. (**C**) Tumor weight. (**D**) Ascites volume. (**E**) Cytokine concentration in the ascites fluid. (**F**) Cardiac weight. (**G**) Myocardial maturity (SDS-MYL1). (**H**) Myocardial ROS (4HNE). (**I**) Myocardial cell death (serum Troponin T levels). (**J**) Autophagy (LC3B). (**K**) Cardiac function (serum BNP). Statistical significance was calculated using an ordinary ANOVA. N, no tumor; C, control; BBR, berberine; HMGB1, high-mobility group box-1; TNF, tumor necrosis factor; SDS-MYL1, sodium dodecyl sulfate-soluble myosin light chain-1; 4HNE, 4-hydroxynonenal; LC3, microtubule-associated protein 1A/1B-light chain 3-B; BNP, brain natriuretic peptide.

Finally, we investigated the impact of BBR on cardiomyocytes in a rat cachexia model (Figure 7). We induced a cachexia model by inoculating rat CRC cells RCN9 into the peritoneal cavity of syngeneic F344 male rats (Figure 7A) [17]. The body weight decreased in the tumor-bearing rat group, but in the BBR group, it recovered to the same level as the tumor-free group (Figure 7B). Notably, the tumor weight decreased by two-thirds (Figure 7C), and the ascites nearly disappeared (Figure 7D). Serum HMGB1, which increased in tumor-bearing rats, was reduced to one-tenth with BBR treatment, while TNFα reduced by one-third (Figure 7F). BBR also restored heart weight and myocardial maturity to normal levels (Figure 7F,G). Additionally, myocardial 4-hydroxynonenal (4HNE), serum troponin T, and myocardial microtubule-associated protein 1A/1B-light chain 3-B (LC3B) (ELISA), which were elevated in tumor-bearing rats, were normalized with BBR treatment (Figure 7H–J). Moreover, serum brain natriuretic peptide (BNP) (ELISA), a marker of heart failure elevated in cancer cachexia, decreased with BBR treatment (Figure 7K). These findings indicate that BBR exerts cardioprotective effects against cancer-induced myocardial damage.

## 3. Discussion

This study sheds light on the role of HMGB1 derived from CRC cells in inducing autophagy and apoptosis in cardiomyocytes, thereby contributing to cancer-related myocardial damage. Moreover, our findings demonstrate the cardioprotective effects of BBR, which suppresses HMGB1 expression. HMGB1 has been implicated in inducing autophagy in skeletal muscles, leading to sarcopenia [20,21,38]. Acting via receptors RAGE and TLR4, HMGB1 triggers NFκB activation, which, as observed in our study, promotes autophagy. Autophagy in skeletal muscle degrades muscle proteins and leads to sarcopenia [20,39]. While moderate autophagy is known to mitigate myocardial damage by eliminating abnormal mitochondria [40,41,42], our data suggest a decrease in mtVol with increasing autophagy, implying suppressed mitochondrial turnover. This phenomenon may be attributed to the reduced expression of peroxisome proliferator-activated receptor γ coactivator 1-α, a key factor in mitochondrial biogenesis [43].

In this investigation, HMGB1 was identified as a key inducer of myocardial apoptosis, aligning with previous research highlighting its involvement in autophagy and apoptosis [44]. While HMGB1-mediated autophagy typically serves as a protective mechanism against apoptosis in cancer cells [45], our study reveals a contrasting effect in the myocardium, where HMGB1 activates pathways such as HMGB1/TLR4/NFκB and HMGB1/extracellular signal-regulated kinase/ETS1, ultimately leading to apoptosis [46,47]. Notably, the HMGB1/RAGE pathway induces both apoptosis and autophagy in the myocardium, contributing to cellular damage [48]. Thus, HMGB1 emerges as a critical mediator of myocardial damage in cancer. Our investigation further demonstrated that BBR effectively mitigated cancer-related myocardial damage by promoting the expression of miRNAs targeting HMGB1. The interaction between BBR and miRNAs constitutes a key mechanism underlying BBR’s antitumor properties [49]. However, the precise mechanism by which BBR modulates miRNAs remains incompletely understood. It has been proposed that BBR may influence miRNA levels by modulating the expression of DNA methyltransferases that epigenetically regulate miRNA expression or by interacting with long non-coding RNAs that sequester miRNAs [50,51]. Specifically, our findings reveal that miR-340-5p and miR-281c-5p effectively suppress HMGB1 expression. These miRNAs have been associated with the suppression of tissue inflammation and the reduction of myocardial and neural damage following ischemia [36,37].

HMGB1 plays a multifaceted role in the progression of various diseases, including its involvement in promoting malignant tumor phenotypes, suppressing anti-tumor immunity by hampering the monocyte system, and triggering an inflammatory cytokine storm in sepsis [19,52]. Targeting HMGB1 through miRNAs has emerged as a promising therapeutic approach, with BBR highlighted as a key candidate. Our study unveils the cardioprotective effects of BBR while also showcasing its tumor-suppressive properties. Notably, BBR not only mitigated tumor growth and reduced ascites in rats but has also demonstrated anticancer and anticancer stem cell effects in CRC [30]. Ferroptosis, closely linked to excessive autophagy, has been implicated in these mechanisms [30]. Intriguingly, disparities in autophagy between cancer cells and cardiomyocytes have been observed. Cancer cells exhibit anomalies in mitochondrial DNA [53] and mitochondrial iron deposition [30], distinct from normal cellular behavior. Consequently, BBR emerges as a dual-action agent, offering both antitumor effects and myocardial protection, thus warranting further clinical investigation.

The most difficult aspect of the clinical application of BBR is its low bioavailability [54]. Only 0.5% of administered BBR is absorbed from the intestinal tract, dropping to 0.35% in the peripheral circulation. Currently, BBR is often administered orally at a dose of about 1 g/body [55]. The effective dose of berberine supplements in humans is reported to be 10 mg/kg [56]. In order to administer the equivalent berberine to rats in drinking water, a concentration of 48 μg/mL was used. It is noteworthy that such a relatively low volume exhibited myocardial protection. Our study did not involve higher doses of BBR or improved delivery [54]. However, the fact that BBR administered in a conservative manner showed myocardial protection suggests that BBR has high clinical value. Further extensive clinical investigation is warranted.

## 4. Materials and Methods

### 4.1. Cell Lines and Reagents

The RCN9 rat colon carcinoma and H9c2 rat cardiomyoblast cell lines were procured from the American Type Culture Collection (Manassas, VA, USA). Cells were maintained in Dulbecco’s modified Eagle’s medium (DMEM) supplemented with 10% fetal bovine serum at 37 °C in a 5% CO_2_ atmosphere. Berberine (BBR) (10 μM for 48 h unless stated otherwise, Tokyo Chemical Industry Co., Ltd., Tokyo, Japan) was obtained from the listed manufacturers. For cytokine neutralization, antibodies against HMGB1 (anti-HMGB1, Biolegend, San Diego, CA, USA), TNFα (anti-rat TNFα, Santa Cruz Biotechnology, Santa Cruz, CA, USA), and IL6 (anti-rat IL6, Abcam, Waltham, MA, USA) were utilized at a concentration of 0.5 μg/mL.

### 4.2. In Vitro Cachexia Model

RCN9 rat colon cancer cells were inoculated into the peritoneal cavity of syngeneic F344 rats, and H9c2 cells (1 × 10^7^ in a 10 cm-culture dish) were treated for 48 h with culture medium containing the ascites mixed with a regular medium at a concentration of 25% (*v/v*). As a control, a culture medium prepared by culturing H9c2 cells (1 × 10^7^ in a 10 cm-culture dish) for 3 days and mixing it with a regular medium at 25% (*v*/*v*) was utilized.

### 4.3. Cell Growth, Cell Death and Apoptosis

Cell growth was assessed using the 3-(4,5-dimethylthiazol-2-yl)-5-(3-carboxymethoxyphenyl)-2-(4-sulfophenyl)-2H-tetrazolium (MTS)-based Celltiter 96 aqueous one-solution cell proliferation assay kit (Promega Corporation, Madison, WI, USA). The absorbance of each well was measured at 490 nm using a Multiscan FC microplate photometer (Thermo Fisher Scientific, Tokyo, Japan). Dead cells were detected using trypan blue staining (Gibco, Thermo Fisher Scientific, Tokyo, Japan), and 1000 cells were observed using light microscopy (Olympus BX43, Tokyo, Japan). Apoptotic cells were detected by Hoechst 33342 staining (Thermo Fisher, Tokyo, Japan) with observation of 1000 cells using an all-in-one fluorescence microscope (KEYENCE, Osaka, Japan).

### 4.4. Mitochondrial Imaging

Mitochondrial function was examined using fluorescent probes. After treatment with or without BBR (25 μM), cells were incubated with the probes for 30 min at 37 °C and photographed using an all-in-one fluorescence microscope (KEYENCE). We employed OxiORANGE (mtHR) (10 μM, Goryo Chemicals, Sapporo, Japan) to assess oxidative stress, mitoGreen (100 nM, PromoCell GmbH, Heidelberg, Germany) to assess mtVol, tetramethylrhodamine ethyl ester (TMRE) (200 nM, Sigma-Aldrich, St. Louis, MO, USA) to assess MMP, and the mitophagy Detection Kit (Dojindo, Kumamoto, Japan) as per the manufacturer’s instructions.

### 4.5. Enzyme-Linked Immunosorbent Assay (ELISA) and Fluorometric Assay

Whole-cell lysates and mitochondrial fractions were prepared as previously described using a RIPA buffer containing 0.1% SDS (Thermo Fisher, Tokyo, Japsn). Protein assays were conducted using the Protein Assay Rapid Kit (Wako Pure Chemical Corporation, Osaka, Japan). The extracted proteins underwent ELISA following the manufacturer’s instructions. The ELISA kits utilized in this study are detailed in Table 1. 

### 4.6. Western Blot

For the preparation of whole-cell lysates, cells were washed twice with cold PBS and harvested. The cells were then lysed using a RIPA buffer containing 0.1% NP-40 (Thermo Fisher, Tokyo, Japan). Protein assays were conducted using the Protein Assay Rapid Kit (Wako, Osaka, Japan). The Minute Cytoplasmic and Nuclear Extraction Kit (Invitrogen, Biotechnologies, Plymouth, MN, USA) was employed for the extraction of nuclear proteins. Protein lysates (25 μg) were separated on 12.5% sodium dodecyl sulfate-polyacrylamide gels, followed by electrotransfer onto a nitrocellulose filter. The membranes were subsequently incubated with primary antibodies and peroxidase-conjugated IgG antibodies (Agilent Technologies, Santa Clara, CA, USA). Immune complexes were detected utilizing an ECL western blot detection system (Amersham, Aylesbury, UK). The primary antibodies used in this analysis are provided in Table 1, employed at a dilution of 1:1000 for immunoblot analysis.

### 4.7. Cell Death Inhibitor Assay

For the cell death inhibitor assay, H9c2 cells were treated with human recombinant HMGB1 (40 μg/mL, Biolegend, San Diego, CA, USA) for 48 h, with or without the addition of cell death inhibitors. The following inhibitors were utilized: N-acetyl-L-cysteine (NAC, 1 mM, Sigma, St. Louis, MO, USA), Z-VAD-FMK (ZVAD, 20 μM, Santa Cruz Biotechnology, Santa Cruz, CA, USA), ferrostatin-1 (FRS, 2 μM), deferoxamine (DFO, 200 μM, Cayman Chemicals, Ann Arbor, MI, USA), and 4-phenylbutyric acid (4PBA, 200 μM, WAKO). Additionally, chloroquine (100 µM, WAKO) was employed to inhibit autophagy.

### 4.8. Reverse Transcription–Polymerase Chain Reaction

Total RNA (0.5 µg) extracted from the three cell lines was subjected to a reverse transcription-polymerase chain reaction (RT-PCR) using the RNeasy kit (Qiagen, Germantown, MD, USA) to evaluate human and murine mRNA expression. The primer sets utilized are detailed in Table 1 and were synthesized by Sigma Genosys (Ishikari, Japan). PCR products were electrophoresed on a 2% agarose gel and stained with ethidium bromide, with ACTB mRNA serving as the internal control.

### 4.9. Small Interfering RNA

Stealth Select RNAi (siRNA) targeting rat HMGB1 and TLR4 were procured from Sigma-Aldrich, St. Louis, MO, USA. AllStars Negative Control siRNA (Qiagen, Valencia, CA, USA) served as the control. The cells were transfected with 10 nM siRNA using Lipofectamine 3000 (Thermo Fisher, Tokyo, Japan), following the manufacturer’s recommendations.

### 4.10. Detection of miRNA

MiRNAs were extracted using the mirVana™ miRNA isolation kit, following the manufacturer’s protocol (Thermo Fisher, Tokyo, Japan). To quantify miRNA expression levels, RT-PCR was conducted using the TaqMan miRNA reverse transcription kit (Applied Biosystems, Thermo Fisher, Tokyo, Japan) and Pri-miRNA Assay kit (Hs04225959_pri, Applied Biosystems), as per the manufacturer’s protocols. The primer sets utilized are detailed in Table 1.

### 4.11. Animals

F344 male rats (5 weeks old, 5 rats per group, SLC Japan, Shizuoka, Japan) were housed in a pathogen-free animal facility under a 12-h light/dark cycle at a temperature of 22 °C and humidity-controlled environment. From the viewpoint of animal welfare, the number of animals used was kept to the minimum that would allow a statistically significant difference to be obtained [57]. Their weight was 133 ± 7 g. All procedures were conducted following institutional guidelines approved by the Committee for Animal Experimentation of Nara Medical University, Kashihara, Japan, in accordance with the current regulations and standards of the Japanese Ministry of Health, Labor and Welfare (approval numbers: 12924, 12925, 23 October 2020). Animals were acclimated to their housing for seven days before the start of the experiment. For the peritoneal dissemination tumor model, RCN9 cancer cells (1 × 10^7^ in 0.2 mL per rat) were injected into the rat peritoneal cavity. Tumor weight was measured by euthanizing the rats on day 22, excising the tumors, and dissecting the peritoneal tumors from non-tumor tissues in the intestine, mesenterium, diaphragm, and abdominal wall. BBR was diluted with distilled water to achieve a final concentration of 48 μg/mL [56]. The solutions underwent ultrasonication for 1 h and vortexing for 30 min. The BBR solution was administered ad libitum, with intake calculated from the amount of water consumed, resulting in a dosage of 3.2 mg/kg body weight/day.

### 4.12. Statistical Analysis

Statistical significance was determined using a two-tailed Fisher’s exact test and an ordinary ANOVA with InStat software (version 3.1; GraphPad, Los Angeles, CA, USA). A significance level of *p* < 0.05 was considered statistically significant.

## Figures and Tables

**Table 1:** Primer sets, antibodies, and ELISA kits.

PCR Primer			
Gene Symbol	Gene Bank ID	Forward Primer (5′-3′)	Reverse Primer (5′-3′)
miR-181c-5p	NR_031897.1	agaacttgccaagggtttg	gcagttccaggcctcggg
miR-340-5p	NR_106669.1	cacttgtactcggtgtga	taagataccaggtatggc
U6	K00784.1	gtgcctgct tcggcagca	cgcttcacgaatttgcgtg
rat HMGB1	NM_012963.3	cacaagaagaagcacccgga	catcctcctcgtcgtcttcc
rat ACTB	NM_031144.3	tcaacaccccagccatgtac	aatgcctgggtacatggtgg
**Antibody**			
**Target**	**Clone or Cat#**	**Company**	
ATG5	EPR1755(2)	Abcam, Waltham, MA, USA	
Beclin1	Clone 669922	R&D Systems, Minneapolis, MN, USA	
LC3	ab51520	Abcam, Waltham, MA, USA	
β-actin	ab8227	Abcam, Waltham, MA, USA	
PARP	GTX100573	GeneTex, Irvine, CA, USA	
HMGB1	3E8	Biolegend, San Diego, CA, USA	
rat TNFα	52B83	Santa Cruz Biotechnology, Santa Cruz, CA, USA	
rat IL6	1.2-2B11-2G10	Abcam, Waltham, MA, USA	
PI3K	#4292	Cell Signaling, Danvers, MA, USA	
AKT	ab8805	Abcam, Waltham, MA, USA	
pAKT	ab38449	Abcam, Waltham, MA, USA	
RAGE	EPR21171	Abcam, Waltham, MA, USA	
TLR4	76B357.1	Abcam, Waltham, MA, USA	
IκBα	66418-1-Ig	Proteintech, Rosemont, IL, USA	
pIκBα	14D4	Cell Signaling, Danvers, MA, USA	
NFκBp65	D14E12	Cell Signaling, Danvers, MA, USA	
Lamin	ab65986	Abcam, Waltham, MA, USA	
BCL2	26593-1-AP	Proteintech, Rosemont, IL, USA	
BAX	E63	Abcam, Waltham, MA, USA	
**ELISA**			
**Target**	**Catalog#**	**Company**	
HMGB1	LS-F4039	Shino-Test, Sagamihara, Japan	
rat TNFα	ab100785	Abcam, Waltham, MA, USA	
rat IL6	ab234570	Abcam, Waltham, MA, USA	
MYL1	orb1211541	Biorbyt, Cambridge, UK	
4HNE	ab238538	Abcam, Waltham, MA, USA	
Troponin T	ABIN6970886	antibodies-online.com, Limerick, PA, USA	
LC3B	#35172	Cell Signaling, Danvers, MA, USA	
rat BNP	ab108816	Abcam, Waltham, MA, USA	

## Data Availability

Data is contained within the article.

## Abbreviations

HMGB1, high-mobility group box-1; RAGE, advanced glycation end products; TLR4, Toll-like receptor-4; CRC, colorectal cancer; BBR, berberine; mtHR, mitochondrial hydroxyl radical; MMP, mitochondrial membrane potential; mitVol, mitochondrial volume; TNFα, tumor necrosis factor-α; IL6, interleukin-6; SDS-MYL1, SDS-soluble myosin light chain 1; NAC, N-acetyl cysteine; PI3 K, phosphoinositide 3-kinases; NFκB, nuclear factor-κB; BCL2, B-cell/CLL lymphoma 2; BAX, Bcl-2-associated X protein; 4HNE, 4-hydroxynonenal; BNP, brain natriuretic peptide.
